# Sleep disorders among health care workers practicing in emergency department in south tunisia

**DOI:** 10.1192/j.eurpsy.2021.1480

**Published:** 2021-08-13

**Authors:** S. Bader, W. Abbes, I. Sellami, W. El Falah, M. Hajjaji, L. Ghanmi

**Affiliations:** 1 Psychiatry, regional hospital of Gabed, gabes, Tunisia; 2 Occupational Medecine, university hospital Hédi Chaker-Sfax, Tunisia, Sfax, Tunisia; 3 The Department Of Psychiatry, Hospital of gabes, Gabes, Tunisia

## Abstract

**Introduction:**

Sleep disorders are the most common health problem among the health care staff, mainly those who perform night shifts.

**Objectives:**

To assess the prevalence of sleep disorders among health care workers in emergency department and to determine its associated factors.

**Methods:**

It was a cross-sectional study, including health care workers assigned to emergency ward and intensive care unit of Hedi Chaker and Habib Bourguiba hospitals in Sfax and regional hospital of Kebili, during the first six months of 2017. We used an anonymous and confidential self-administered questionnaire. We used hospital anxiety and depression scale (HAD) to assess anxiety and depression. Sleep quality was assessed by the Pittsburgh Sleep Quality Index and day time sleepiness by the Epworth Sleepiness Scale.

**Results:**

240 nurses were included. Mean age was 37 years-old, 59.2% were female and 64.2% were married and 79.2% assured night shifts. The prevalence of sleep disorders was 70.4%. Sleep difficulties were significantly correlated with anxiety (p=0.001) and depression (p=0.02). In multivariate study, sleep disorders were related to the absence of leisure activity (OR=0.42 [0.19-0.94]; p=0.035) and anxiety (OR=3 [1.4-6.1]; p=0.002). 40.8% of nurses experienced drowsiness. Sleepiness was significantly correlated with the absence of leisure activities (p=0.04) and with psychiatric family history (p=0.02). In the multivariate study, sleep disorders were correlated with female gender (OR=0.43 [0.19-0.9]; p=0.042) and with no leisure activity (OR=2.6 [1.2-5.6]; p=0.01).

**Conclusions:**

Sleep disorders were common among emergency nurses, in order of that; working conditions should be improved to provide less stressful conditions for nurses.

**Conflict of interest:**

No significant relationships.

